# Brachytherapy dose‐volume histogram commissioning with multiple planning systems

**DOI:** 10.1120/jacmp.v15i2.4620

**Published:** 2014-03-06

**Authors:** Michael S. Gossman, Samuel S. Hancock, Rajat J. Kudchadker, Paul R. Lundahl, Minsong Cao, Christopher S. Melhus

**Affiliations:** ^1^ Radiation Oncology Department Tri‐State Regional Cancer Center Ashland KY; ^2^ Regulation Directive Medical Physics Flatwoods KY; ^3^ Radiation Oncology Department Southeast Missouri Hospital Cape Girardeau MO; ^4^ Department of Radiation Physics UT MD Anderson Cancer Center Houston TX; ^5^ Radiation Oncology Department Riverside Methodist Hospital Columbus OH; ^6^ Radiation Oncology Department UCLA School of Medicine Los Angeles CA; ^7^ Radiation Oncology Department Tufts University School of Medicine Boston MA USA

**Keywords:** brachytherapy, dose, DVH, histogram, volume

## Abstract

The first quality assurance process for validating dose‐volume histogram data involving brachytherapy procedures in radiation therapy is presented. The process is demonstrated using both low dose‐rate and high dose‐rate radionuclide sources. A rectangular cuboid was contoured in five commercially available brachytherapy treatment planning systems. A single radioactive source commissioned for QA testing was positioned coplanar and concentric with one end. Using the brachytherapy dosimetry formalism defined in the AAPM Task Group 43 report series, calculations were performed to estimate dose deposition in partial volumes of the cuboid structure. The point‐source approximation was used for a ${\;}^{125}I$ source and the line‐source approximation was used for a ${\;}^{192}\text{Ir}$ source in simulated permanent and temporary implants, respectively. Hand‐calculated, dose‐volume results were compared to TPS‐generated, dose‐volume histogram (DVH) data to ascertain acceptance. The average disagreement observed between hand calculations and the treatment planning system DVH was less than 1% for the five treatment planning systems and less than 5% for 1 cm≤r≤5 cm. A reproducible method for verifying the accuracy of volumetric statistics from a radiation therapy TPS can be employed. The process satisfies QA requirements for TPS commissioning, upgrading, and annual testing. We suggest that investigations be performed if the $\text{DVH}\%{\text{Vol}}_{\text{TPS}}$ “actual variance” calculations differ by more than 5% at any specific radial distance with respect to $\%{\text{Vol}}_{\text{TG}-43}$, or if the “average variance” DVH $\text{DVH}\%{\text{Vol}}_{\text{TPS}}$ calculations differ by more than 2% over all radial distances with respect to $\%{\text{Vol}}_{\text{TG}-43}$.

PACS numbers: 87.10.+e, 87.55.‐x, 87.53.Jw, 07.05.Tp

## I. INTRODUCTION

It is the standard of practice for a radiation oncologist to review various brachytherapy plan iterations and approve one as the desired approach for treatment. Patient‐specific plan iterations are generated with a computerized treatment planning system (TPS) using cross‐sectional image sets. Although the imaging data may be from different types of equipment (e.g., a computerized tomography (CT) scanner or ultrasound (US) unit), a dose distribution can be projected onto those images using source‐specific brachytherapy dosimetry parameters. The dose distribution, which is qualitatively represented in the form of isodose lines superimposed on the images, depicts which anatomical part of the patient is estimated to receive a certain dose. Quantitative interpretation of the volumetric dose distribution is necessary to obtain definitive judgment on the acceptable dose to a given designated volume (e.g., organ at risk or target). The radiation oncologist generally utilizes a dose‐volume histogram (DVH) analysis for this quantitative interpretation. Although the DVH carries no spatial information, it can differentiate the percentage of the prescribed dose received to any organ or structure being considered.^(1)^ Clinical interpretation of organ‐specific DVH data is a deciding factor in whether a treatment plan is acceptable.^(2)^ Therefore, DVH metrics reported by TPSs need to be examined routinely to ensure the calculations are correct.

There are no detailed recommendations from the American Association of Physicists in Medicine (AAPM) on how such quality assurance (QA) testing of the DVH should be performed and/or assessed. The AAPM Task Group 53 (TG‐53) report^(3)^ indicates no traditional tolerance for this testing. The report states that DVH validation depends on many factors including the dose calculation grid, volumetric region‐of‐interest grid, accuracy of object segmentation, bin size of the histogram, and any plan normalization/^3,4^ Since it is important to have accurate DVH results for clinical use, we have tasked ourselves to examine the accuracy of the DVH output in the computerized TPS against independent hand calculations.

In our review of published literature, there were no observed publications related to this type of testing found for any brachytherapy system. There have been several publications citing the need for DVH QA.^5^, ^6^, ^7^, ^8^, ^9^ However, other than providing the suggestion that medical physicists ensure that computerized DVH analysis data are correct, none have provided a quantitative QA test for brachytherapy TPS DVHs.

In 2010, Gossman and Bank^(10)^ comprehensively described an independent DVH QA process for particle accelerator‐based treatments. In that process, a narrow, rectangular cuboid structure with extended length parallel to the beam orientation was employed. The fractional cuboid volume receiving a fraction of the prescribed dose was shown to be directly related to the dose calculated to a particular depth along the cuboid.^(9)^ We have chosen to modify the technique towards an inexpensive, routine, simple, and accurate QA strategy that can be applied to brachytherapy TPSs. To our knowledge, this is the first independent QA process for DVH validation when employing a sealed brachytherapy source. In this analysis, we provide DVH QA results for multiple TPSs using identically characterized low‐dose rate (LDR) and high‐dose rate (HDR) radionuclide sources.

## II. MATERIALS AND METHODS

### A. Brachytherapy dose calculations

The AAPM Task Group 43 (TG‐43) report from 1995 and updates TG‐43U1 and TG‐43U1S1 are currently recognized as the worldwide standard for low‐energy, photon‐emitting brachytherapy dose calculations.^11^, ^12^, ^13^ The high‐energy, photon‐emitting brachytherapy dosimetry protocol is described by the joint AAPM/ESTRO High Energy Brachytherapy Dosimetry Working Group Report.^(14)^ A detailed description of the formalism involved is available in that literature. The general form for the calculation of absorbed dose in a permanent implant for the point‐source approximation is given in Eq. (1).^(11)^

(1) $$D(r)=S_{k}\cdot \Lambda \cdot {\left(\frac{r_{0}}{r}\right)}^{2}\cdot g_{p}(r)\cdot \varphi_{an}(r)\cdot \tau$$

In this equation, the initial dose rate is converted to total dose when multiplying by the mean lifetime, τ. The mean lifetime is defined as $\tau =1.44\times t_{1/2}$, where $t_{1/2}$ represents the half‐life.

The general form for the calculation of absorbed dose using the line‐source approximation is given in Eq. (2) for a temporary implant of duration (t).^(11)^

(2) $$D(r)=S_{K}\cdot \Lambda \cdot \left(\frac{G_{L}(r,\theta )}{G_{L}(r_{0},\theta_{0})}\right)\cdot g_{L}(r)\cdot F(r,\theta )\cdot t$$

The geometry function $\left(G_{L}(r,\theta )\right)$ approximates the intensity fall‐off due to distance (i.e., inverse square law effects for a distributed line source). The ratio of the two geometry functions details the difference in the angular and radial position of the source between the reference position and point‐of‐interest geometry. It can be shown that the ratio of geometry functions used in Eq. (2) reduces to a simplified form, since $\left(r_{0},\theta_{0}\right)$ represent the reference position at $\left(r=1\;\text{cm},\theta =90^{\circ }\right)$. The reduced form for this ratio is shown in Eq. (3). This form may be used to simplify the hand calculations needed.

(3) $$\left(\frac{G_{L}(r,\theta )}{G_{L}(r_{0},\theta_{0})}\right)=\left(\frac{r_{0}\cdot {\text{tan}}^{-1}\left(\frac{L}{2r}\right)}{r\cdot {\text{tan}}^{-1}\left(\frac{L}{2r_{0}}\right)}\right)$$

Commercially available TPSs implement these dose calculation methods in subtly different ways. Specific differences are outlined in the Materials and Methods section B.

#### A.1 LDR ${\;}^{125}I$ source modeling

The Model 6711 (GE Healthcare & Oncura, Inc.; Arlington Heights, IL) sealed source was chosen for use in LDR DVH verification. Brachytherapy dosimetry parameters used in the point‐source approximation are those detailed in the updated AAPM TG‐43U1 report.^12^, ^13^ Specific parameters employed include a dose‐rate constant (Λ) of $0.965\;\text{cGy}\;h^{-1}U^{-1}$, radial dose function $\left(g_{P}(r)\right)$ taken in 1 cm increments, and mean lifetime (τ) of 2053 h.^(12)^ The 1D anisotropy function $\left(\varphi_{an}(r)\right)$ was set to unity in all calculations, due to limited data entry available in specific TPSs. Source strength of 1.01 U was assigned to deliver a permanent implant dose of 20 Gy at the reference position. For the point‐source approximation, source orientation does not matter.

#### A.2 HDR ${\;}^{192}\text{Ir}$ source modeling

Two different HDR sources were studied. The VariSource Model VS2000 (Varian Medical Systems, Inc.; Palo Alto, CA) was commissioned using published brachytherapy dosimetry parameters from Angelopoulos et al.^(15)^ Source strength of 27,292 U was assigned for a treatment time of 20 min (0.333 h), which yields a prescription dose of 100 Gy at the reference position. The dose‐rate constant, Λ, was $1.101\;\text{cGy}\;h^{-1}U^{-1}$, the geometry function used an effective length of 0.5 cm, and the 2D anisotropy function (F(r,θ)) was set to 1.00 for r and θ to simplify the hand calculation. The Model mHDR‐v2 (Nucletron‐Elekta; Stockholm, Sweden) source was also commissioned for use.^15^, ^16^ As done for the Model VS2000, a source strength of 27,076 U was selected to deliver 100 Gy at the reference position from a 20 min temporary implant. The dose rate constant for the mHDR‐v2 was $1.108\;\text{cGy}\;h^{-1}U^{-1}$, the active length was 0.36 cm, and all other dosimetry parameters were taken from Daskalov et al.^17^, ^18^ The 2D anisotropy function was set to unity over r and θ. For the line‐source approximation used in HDR source modeling, source placement and orientation must be done identically for a consistent solution.

### B. Treatment planning systems

Dose calculations were performed in several different brachytherapy TPSs for DVH evaluation. While this investigation wasn't designed to compare all commercially available TPSs, at least two TPSs were used for each source model investigated. In each TPS, a narrow rectangular cuboid having dimensions $0.5\;\text{cm}\times 0.5\;\text{cm}\times 5.0\;\text{cm}\;(1.25\;{\text{cm}}^{3})$ was delineated on various planning images. Brachytherapy source models were assigned to the center of one $0.5\times 0.5\;{\text{cm}}^{2}$ edge of the cuboid. For each plan, the source axis was perpendicular to the long axis of the cuboid to ensure consistent application of the line‐source approximation for 2D calculations by the TPS. Percentage isodose values computed by the TPS extended outward and around the source. At each distance from the source there existed a different isodose line passing through a measureable length along the cuboid. A partial volume can be associated to the relative dose at that distance and is provided in the form of a DVH by the TPS. These data were tabulated for comparison to hand‐calculated results. Each TPS was commissioned indistinguishably for the brachytherapy source intended for planning, including the consideration that some TPSs prohibit use of the anisotropy function for the point‐source approximation.

TPS‐specific considerations and findings are outlined in the following sections. Note that a variety of radiographic images sets or “empty” user‐defined datasets could be employed in the application of this DVH QA process. Specific methods are described below as examples of how the DVH QA process could be applied in various clinical settings.

#### B.1 VariSeed

**Figure 1:**
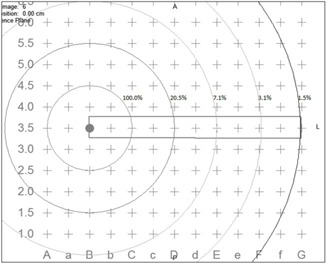
Axial view of the ${\;}^{125}I$ isodose distribution superimposed on the rectangular cuboid as depicted in VariSeed for treatment planning of a permanent implant.

The Varian VariSeed Version 8.0.1 TPS was examined. Planning images were obtained from a General Electric (Fairfield, CT) Model Logic P5 ultrasound station. Software Version R2.0.3 was used with a Model ERB US probe operating at 6‐10 MHz. The stepper was a CR Bard (Salt Lake City, UT) Model Sure‐Point with grid Model D02100‐18 (AaBbCc…). Using a water bath with 3 L sterile irrigation water, the probe was rested in the center of the phantom for acquisition. A total of seven images were captured with that template projection as the stepper transitioned in 0.25 cm increments. Given the assignment of a slice near the centroid of the phantom containing the origin of the scan, a contour was created in multiple slices to form the rectangular cuboid. The central slice was designated the origin. In the axial view, a contour was drawn from grid space B3.25 to G3.25 for a total of 5.0 cm laterally, then anterior to G3.75, laterally to B3.75 and back to B3.25, making for a height of 0.5 cm. The contour was identically drawn on slices immediately superior and inferior for a net contoured width of 0.5 cm in three slices, resulting in a 1.25 cm^3^ volume. The source was assigned to grid space B3.5, which oriented it in the superior‐inferior direction, at precisely the edge of the contour. The VariSeed TPS view is depicted in Fig. 1.

For the DVH evaluation of VariSeed, the Model 6711 was commissioned.

#### B.2 BrachyVision

Varian BrachyVision Version 10.0 was also considered. Planning images were acquired using a virtual computerized tomography phantom option in the Varian Eclipse workspace. The virtual phantom generated was 20 cm×20 cm in 1003 slices, each having a plane separation of 0.1 cm. A pixel density of 512×512 was used, ensuring a pixel resolution of 0.04 cm^2^. In the axial view, the contour began at isocenter and extended 5.0 cm laterally and with a height of 0.5 cm. The object contour was identically drawn on two slices immediately superior and three slices inferior to the slice containing the origin, for a net contoured width of 0.5 cm in six slices. In order to maintain the desired shape of the contour, the option to convert the contour to a high‐resolution object was used. In addition, drawing with the “rectangular tool” using a 0.1 cm grid space was activated. A volume of 1.25 cm^3^ was achieved. A brachytherapy source was placed symmetrically at the central edge of the contour.

For DVH evaluation, the Models 6711 and VS2000 were commissioned. The same planning images were used for both source models.

#### B.3 Pinnacle^3^

Pinnacle^3^ (Philips Healthcare; Amsterdam, Netherlands) Version 8.0M was similarly investigated. A plan was created using the “digitize contours” option, which allows for the specification of spatial voxel dimensions. The slice width (z‐dimension) was set to 0.25 cm, with an in‐plane resolution of 0.1 cm in both the x‐ and y‐axes. A total of 81 slices were created to span from −10 cm to 10 cm with the in‐plane origin, represented as (x, y), at (0.05, 0.05) in centimeters. A narrow rectangular cuboid having dimensions $0.5\times 5.0\;{\text{cm}}^{2}$ was contoured on three consecutive slices using the point‐by‐point contour tool. Subsequently, the region‐of‐interest data file was edited to precisely locate each vertex point at (0,0.25),(0,−0.25),(5,−0.25), and (5, 0.25). Because these vertices were in the center of a voxel, a total volume of 1.88 cm^3^ resulted (three slices×0.25 cm slice thickness×0.5 cm×5 cm). If the same volume is characterized on five 0.1 cm thick slices, then a volume of 1.255 cm^3^ is obtained. A density of $1.0\;{\text{g cm}}^{-3}$ was assigned to the contoured volume because Pinnacle^3^ cannot calculate or display brachytherapy isodose lines in a volume having density below the outside‐patient air threshold. The dose grid resolution was set to 0.2 cm^3^.

Sources were each commissioned using the point‐ and line‐source approximations. Brachytherapy dosimetry parameters were used to calculate a two‐dimensional “look‐up table” of dose at specified along‐away distances. This table was calculated with 0.1 cm^2^ resolution for all three source models.

#### B.4 Symphony

Symphony (MIM Software, Inc.; Cleveland, OH) software Version 5.5.6 LDR prostate brachytherapy TPS was analyzed. A prostate implant CT dataset with 2.5 mm axial slice thickness, resolution of 512×512 pixels and a field of view of 50 cm was imported into MIM for the purpose of DVH testing. Once the image set was successfully imported, a cuboid with dimensions of 0.5 cm×5 cm was contoured on three consecutive slices using a point‐by‐point contouring tool. The resulting total volume was 1.875 cm^3^ over three slices, because Symphony utilizes the full voxel thickness. One ${\;}^{125}I$ Model 6711 seed was positioned at one end of the rectangular cuboid in the middle of the surface, as in Fig. 1. A dose grid of 0.2 cm^3^ was used for calculation of dose distributions.

#### B.5 Oncentra

The Nucletron Model Oncentra TPS version 4.1 SP2 was evaluated using the ${\;}^{192}\text{Ir}$ Model mHDR−v2 source. A slice thickness of 0.1 cm in an idealized cubic virtual patient was used. The calculation grid spacing was 0.1 cm^3^. All defining positions of the structure, source, and dose point were entered with exact coordinates. Oncentra reported the volume of the structure to be 1.19 cm^3^. The Oncentra TPS limits the entry and modification of source brachytherapy dosimetry parameter data. As such, F(r,θ) data were not set to unity for DVH calculations and were included in the calculation.

III. RESULTS

Table 1 provides the parameters and calculations involved for the determination of dose to five distances from the Model 6711 ${\;}^{125}I$ source. With a contoured cuboid structure length of 5.0 cm, it follows that for every 1.0 cm away from the source, the dose delivered at that point incrementally envelopes approximately 20% of the cuboid structure. The result enables tabulation of $\%{\text{Vol}}_{TG-43}$. Subtle differences in the variation of r across the 0.25 cm cuboid width were ignored, as differences become negligible with increasing r. The calculations were designed to deliver 100% of the prescription dose at r=1.0 cm, as denoted by $\%{\text{Dose}}_{TG-43}$.

As shown for particle accelerator treatments,^(10)^ the isodose distribution for external beam radiation is not perfectly flat. Off‐axis factors in the open beam profile can be used as a correction factor to the volume covered at any specific isodose line.^(10)^ We understood that the isodose distribution at a constant distance from a radioactive source is similarly not constant. At each radial distance of interest there exists a rounded dose distribution that slightly reduces the covered cuboid volume from the ideal partial volumes of 20%, 40%, 60%, 80%, and 100%.

A correction factor can similarly be applied to account for this coverage reduction. This factor can be directly calculated using the source‐specific radial dose function and 1D‐ or 2D‐geometry functions. Alternatively, a simple correction factor can be determined based on isotropic source falloff. In our hand calculations, correction factors were determined based on a perfect circle, centered about the point source. Volumetric correction factors determined geometrically by the known sagitta and apothem were found to be 1.045, 1.021, 1.014, 1.010, and 1.008 for radial distances 1 cm, 2 cm, 3 cm, 4 cm, and 5 cm, respectively. Reducing the volumes accordingly, $\%{\text{Vol}}_{TG-43}$ at each distance r then becomes 19.1%, 39.2%, 59.2%, 79.2%, and 99.2%. Calculations for each source considered are presented using these isotropic correction factors.

The percentage relative dose received to partial volumes of the structure contoured in the TPSs was taken directly from each of the corresponding DVHs. The cumulative results for hand calculations are listed in 1, 3. DVH data plots were then combined and reproduced from all TPSs and hand calculations. The general form of the plot resembling a decaying exponential or power reduction was expected. This is evident from 2, 4 for the model 6711, VS2000, and mHDR‐V2, respectively, with embedded results for each specific TPS.

**Table 1:** Parameters and results for hand calculations of DVH statistics using the ${\;}^{125}I$ Oncura Model 6711 source. Constants used in the calculation include: $S_{K}(U)=1.01,\Lambda (\text{cGy}/U-h)=0.965,\phi_{\text{an}}(r)=1$, and τ(h)=2053

*r (cm)*	$(r_{0}/{r)}^{2}$	$g_{p}(r)$	*D(Gy)*	$\%Vol_{TG-43}$	$\%Dose_{TG-43}$
1	1.000	1.000	20.01	19.1	100.0
2	0.250	0.819	4.10	39.2	20.5
3	0.111	0.636	1.41	59.2	7.1
4	0.063	0.499	0.62	79.2	3.1
5	0.040	0.367	0.29	99.2	1.5

**Table 2:** Parameters and results for hand calculations of DVH statistics using the ${\;}^{192}\text{Ir}$ Varian Model VS2000. Constants used in in the calculation include: $S_{K}(U)=27,292,\Lambda (\text{cGy}/U-h)=1.101,F(r,\theta )=1$, and t(h)=0.33

*r (cm)*	$G_{L}(r,\theta )/G_{L}(r_{0},\theta_{0})$	$g_{L}(r)$	*D(Gy)*	$\%Vol_{TG-43}$	$\%Dose_{TG-43}$
1	1.000	1.000	100.16	19.1	100.0
2	0.254	1.005	25.55	39.2	25.5
3	0.113	1.006	11.40	59.2	11.4
4	0.064	1.002	6.39	79.2	6.4
5	0.041	0.993	4.06	99.2	4.1

TPS computed dose volume results were generally consistent with hand calculations. The results for TPSs with respect to TG‐43 formalism are shown in Table 4. As for the discussion above, %Vol refers to the percentage of partial volume covered by the isodose line at each %Dose listed.

The average results for all systems at all distances and for all sources were within ±1%. No TPS calculated differences exceeding ±5% at specific points of analysis. Overall, every TPS showed minimal deviations from hand calculations and each produced acceptable results.

**Table 3:** Parameters and results for hand calculations of DVH statistics using the ${\;}^{192}\text{Ir}$ Nucletron Model mHDR‐v2. Constants used in the calculation include: $S_{K}(U)=27,076,\Lambda (\text{cGy}/U-h)=1.108,F(r,\theta )=1$, and t(h)=0.33

*r (cm)*	$G_{L}(r,\theta )/G_{L}(r_{0},\theta_{0})$	$g_{L}(r)$	*D(Gy)*	$\%Vol_{TG-43}$	$\%Dose_{TG-43}$
1	1.000	1.000	100.16	19.1	100.0
2	0.256	1.007	25.83	39.2	25.8
3	0.115	1.008	11.55	59.2	11.5
4	0.065	1.004	6.48	79.2	6.5
5	0.041	0.995	4.11	99.2	4.1

**Figure 2:**
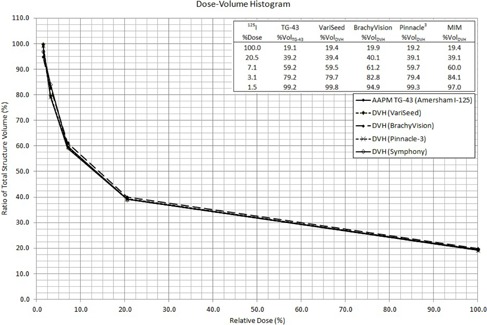
DVH for all methods in treatment planning with the Oncura ${\;}^{125}I$ Model 6711.

**Figure 3:**
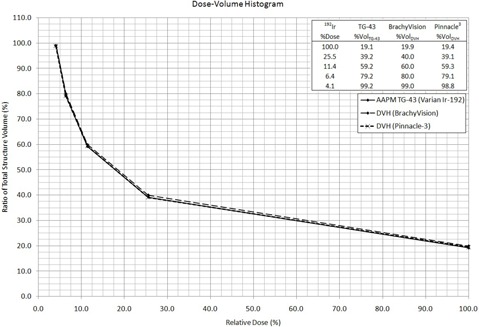
DVH for all methods in treatment planning with the Varian ${\;}^{192}\text{Ir}$ Model VS2000

**Figure 4:**
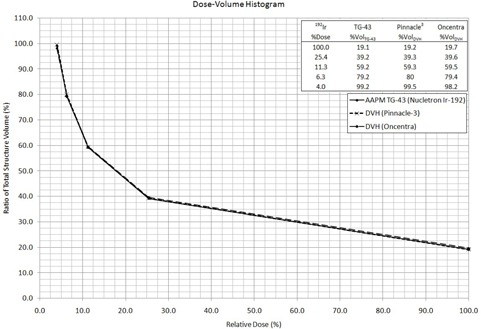
DVH for all methods in treatment planning with the Nucletron ${\;}^{192}\text{Ir}$ Model mHDR‐v2

**Table 4:** Summary of DVH statistics for the three source models and five TPSs, with average values and extreme values summarized for each case. Note that all cases meet the 2% average and 5% individual difference metrics

		*TPS %Vol Difference*				
*Source Model*	*%Dose*	*VariSeed*	*BrachyVision*	*Pinnacle* ^3^	*MIM*	*Oncentra*
6711	100.0	0.3	0.8	0.1	0.3	
	20.5	0.2	0.9	−0.1	−0.1	
	7.1	0.3	2.0	0.5	0.8	
	3.1	0.5	3.6	0.2	4.9	
	1.5	0.6	−4.3	0.1	−2.2	
	**Average:**	0.4	0.6	0.2	0.7	
	**Range:**	(0.2,0.6)	(−4.3,3.6)	(−0.1,0.5)	(−2.2,4.9)	
VS2000	100.0		0.8	0.3		
	25.5		0.8	−0.1		
	11.4		0.8	0.1		
	6.4		0.8	−0.1		
	4.1		−0.2	−0.4		
	**Average:**		0.6	0.0		
	**Range:**		(−0.2,0.8)	(−0.4,0.3)		
mHDR‐v2	100.0			0.1		−0.6
	20.5			0.1		−0.4
	7.1			0.1		−0.3
	3.1			0.8		−0.2
	1.5			0.3		1.0
	**Average:**			0.3		−0.1
	**Range:**			(0.1,0.8)		(−0.6,1.0)

## IV. DISCUSSION

Clinical evaluation of brachytherapy DVH data must be made with caution. It is important for clinicians to recognize the limitations of different TPSs and how those limitations might affect DVH results. Some examples are that current post‐implant clinical imaging may not clearly identify source orientation and that some TPSs cannot compute 2D anisotropy.

In addition to clinical considerations, there are TPS‐specific differences in the methods and tools available for contour delineation. As an example, in VariSeed the contoured structure maintains its shape when the mouse is released for that ultrasound image slice. This is not true for BrachyVision, which operates in the Varian Eclipse environment where CT scans are used rather than ultrasound images. In Eclipse, the software did not hold the contour shape very well when the mouse is released. Even attempting to use rectangular contouring tools resulted in markedly inaccurate rounding of the ends. In general, for each attempt at contouring a two‐dimensional structure on any slice, the result will be less area than was intended. This is consistent with the finding of less structural volume covered at any relative dose level for BrachyVision, compared to hand calculations. This is always true if the cross section of the volume varies as a function of the length across the volume. As long as the contour is consistently shaped and symmetric around the source, the volume change per unit length is proportional.

Other software also presented challenges. In Pinnacle^3^ and Symphony, although the chosen rectangular cuboid was to have dimensions $0.5\times 0.5\times 5.0\;{\text{cm}}^{3}(1.25\;{\text{cm}}^{3})$, the resulting volume was 1.88 cm^3^ due to 0.25 cm thick voxels employed in the analyses. This disagreement was found to have no consequence to the overall accuracy of the technique. As an example of TPS‐specific volume estimation, Pinnacle^3^ calculates volume using Eq. (4).

(4) $$V_{ROI}=V_{Voxel}\cdot (N_{Inner}+0.5\;\cdot \;N_{Edge})$$

Here, $V_{ROI}$ is the ROI volume, $V_{Voxel}$ is the voxel volume, $N_{Inner}$ is the number of inner voxels, and $N_{Edge}$ is the number of edge voxels. As such, Pinnacle assumes that the average voxel is bisected by the contour edge, which is likely the case for anatomical contours, but may not be the case for regular geometric structures as used in this study. When the Pinnacle^3^ voxel thickness was reduced to 0.1 cm and the structure defined on five consecutive slices, the resultant ROI volume was in very good agreement with the expected value (0.4% difference). Comparing DVH results using the two voxel sizes demonstrated very small differences in the overall accuracy of the TPSs because DVH analyses compare relative doses to relative volumes. Care should be taken to ensure the highest resolution and accuracy when contouring in order to minimize errors that affect $\%{\text{Vol}}_{\text{TG}-43}$ directly. For example, the evaluation ROI must have a constant cross‐sectional area to ensure proportionality between relative structure volume and distance from the source. Users could seek to employ smaller voxel resolution in TPSs for DVH testing to limit the potential impact of the volume calculation methodology.

Even though BrachyVision prohibits the use of the 1D anisotropy function for the point‐approximation, the DVH validation methodology could be employed. As for some other TPSs, Oncentra was not capable of forcing the anisotropy function to be unity. For Oncentra, the source data were preloaded by the manufacturer. The DVH data required no manipulation since the resulting $\phi_{\text{an}}(r)$ was found to be 1.000±0.005 throughout the rectangular cuboid, which was consistent with the calculation on the perpendicular bisector plane. One should note that the radionuclide or source model of choice is immaterial, as long as consistent brachytherapy dosimetry parameters are applied in the TPS and hand calculations.

For TPSs with the capability of fine resolution contouring, we have considered the use of a square contour with narrow thickness (12 cm×12 cm×0.1 cm). For this alternative investigation, the source may be placed at the centroid of two identical slices. It may be noted that %Vol would then contain the area term denoted $\pi r^{2}$. In this methodology, minor limitations in achieving a thickness of 0.1 cm can be ignored, since this thickness cancels out of the calculation entirely. Test calculations have proven this method to be alternatively acceptable, as well, with less than 5% actual variance and less than 2% average variance. Another option is to calculate by hand the volumetric dose distribution using a spreadsheet. A spreadsheet could be used to calculate the dose to the center of each voxel, where the relative partial volume dose could then be calculated for comparison to TPS results. There is uncertainty in the results of any such testing due to the discrete steps in the DVH curve. For the rectangular cuboid method described here, the steps are nominally 2%. In comparison to using a cuboid, a 1 mm contoured structure with a circular isodose volume may reduce this uncertainty.

## V. CONCLUSIONS

According to the AAPM TG‐53 report, DVH analysis should be conducted at least annually.^(3)^ We have provided a step‐wise process for the creation of an in‐house test to satisfy this requirement in TPSs designed for brachytherapy applications. The methodology incorporates a single radioactive source adjacent and orthogonal to a contoured cuboid structure of known volume. At each distance from the source there existed a different isodose line passing through the corresponding length of the contour, thus relating to a percentage of the volume. This partial volume is associated to the relative dose at that distance as computed by the software, and provided in the form of a point on a DVH curve. These data can be readily evaluated using this published formalism for hand calculations. This process was conducted for multiple TPSs. Using identical methodology, the average disagreement observed between hand calculations and the TPS DVH was less than ±1% for the TPSs studied. The variation at each distance (1 cm≤r≤5 cm) was also within 5% for all data.

We agree that the criterion of the AAPM TG‐53 report to validate the DVH annually for all brachytherapy TPSs should be satisfied.^(3)^ We conclude that since there is no explanation or tolerance provided in that report, that this methodology could be instituted in clinics as the standard of practice. Our suggestion is that if the DVH $\%{\text{Vol}}_{\text{TPS}}$ “actual variance” calculations differ by more than 5% at any radial distance with respect to $\%{\text{Vol}}_{\text{TG}-43}$, or if the DVH $\%{\text{Vol}}_{\text{TPS}}$ “average variance” calculations differ by more than 2% over all radial distances with respect to $\%{\text{Vol}}_{\text{TG}-43}$, that the disparity should be investigated. A reproducible method for verifying the accuracy of volumetric dose data from a radiation therapy treatment planning system can be employed, satisfying the QA requirements for TPS commissioning, upgrades, and annual evaluation, regardless of the TPS, source approximation method, source model, or implant type.

## ACKNOWLEDGMENTS

A portion of this research was presented at the 2012 Fall Symposium organized by the Ohio River Valley Chapter of the American Association of Physicists in Medicine in Indianapolis, IN (by MSG).

## Supporting information

Supplementary Material

Supplementary Material
