# Supplementary material for: Brachytherapy dose‐volume histogram commissioning with multiple planning systems
Source: J Appl Clin Med Phys. 2014 Mar 6;15(2):110–20. doi: 10.1120/jacmp.v15i2.4620 (PMC5875493; doi:10.1120/jacmp.v15i2.4620)
Supplement: Supplementary file 1 — Supplementary Material [file ACM2-15-110-s001.docx]

**Brachytherapy dose-volume histogram commissioning with multiple planning systems**

**^1,2^ Michael S. Gossman, ^3^ Christopher S. Melhus, ^4^ Samuel S. Hancock,
^5^ Rajat J. Kudchadker, ^6^ Paul R. Lundahl, ^7^ Minsong Cao**

*^1^ Tri-State Regional Cancer Center, Radiation Oncology Department, 706 23^rd^ Street, Ashland, KY 41101*

[*mgossman@tsrcc.com*](mailto:mgossman@tsrcc.com)

*^2^ Regulation Directive Medical Physics, 3312 Forestdale Court, Flatwoods, KY 41139*

[*mgossman@tsrcc.com*](mailto:mgossman@tsrcc.com)

*^3^ Tufts University School of Medicine, Radiation Oncology Department, 750 Washington Street # 246, Boston, MA 02111*

[*cmelhus@tuftsmedicalcenter.org*](mailto:cmelhus@tuftsmedicalcenter.org)

*^4^ Southeast Missouri Hospital, Radiation Oncology Department, 789 South Mt. Auburn Road, Cape Girardeau, MO 63703*

[*shancock@sehealth.org*](mailto:shancock@sehealth.org)

*^5^ UT MD Anderson Cancer Center, Department of Radiation Physics, 1515 Holcombe Boulevard, Unit 1202, Houston, TX 77030*

[*rkudchad@mdanderson.org*](mailto:rkudchad@mdanderson.org)

*^6^ Riverside Methodist Hospital, Radiation Oncology Department, 3535 Olentangy Road, Columbus, OH 43214*

[*lundahp@ohiohealth.com*](mailto:lundahp@ohiohealth.com)

*^7^ UCLA School of Medicine, Radiation Oncology Department, 200 UCLA Medical Plaza, Suite B265, Los Angeles, CA 90095*

[caominsong@yahoo.com](mailto:caominsong@yahoo.com)

Corresponding author: Michael S. Gossman, M.S., DABR, FAAPM,

Chief Medical Physicist & RSO

Tri-State Regional Cancer Center

Radiation Oncology Department

706 23rd Street, Ashland, KY 41101

[*mgossman@tsrcc.com*](mailto:mgossman@tsrcc.com)

Running Title: Brachytherapy DVH commissioning
