# Supplementary material for: Brachytherapy dose‐volume histogram commissioning with multiple planning systems
Source: J Appl Clin Med Phys. 2014 Mar 6;15(2):110–20. doi: 10.1120/jacmp.v15i2.4620 (PMC5875493; doi:10.1120/jacmp.v15i2.4620)
Supplement: Supplementary file 2 — Supplementary Material [file ACM2-15-110-s002.doc]

**Brachytherapy dose-volume histogram commissioning with multiple planning systems**

July 10, 2013

Dear Editor-in-Chief,

I attest as the corresponding author for this research article that the material enclosed has never been published and free of copyright from all entities. Please review the manuscript accordingly. It has been authenticated and approved for review by all authors. Thank you kindly.

Sincerely,

Michael S. Gossman, M.S., DABR, FAAPM

^1,2^ Michael S. Gossman, ^3^ Christopher S. Melhus, ^4^ Samuel S. Hancock
^5^ Rajat J. Kudchadker, ^6^ Paul R. Lundahl, ^7^ Minsong Cao

*^1^ Tri-State Regional Cancer Center, Radiation Oncology Department, 706 23^rd^ Street, Ashland, KY 41101*

*^2^ Regulation Directive Medical Physics, 3312 Forestdale Court, Flatwoods, KY 41139*

*^3^ Tufts University School of Medicine, Radiation Oncology Department, 750 Washington Street # 246, Boston, MA 02111*

*^4^ Southeast Missouri Hospital, Radiation Oncology Department, 789 South Mt. Auburn Road, Cape Girardeau, MO 63703*

*^5^ UT MD Anderson Cancer Center, Department of Radiation Physics, 1515 Holcombe Boulevard, Unit 1202, Houston, TX 77030*

*^6^ Riverside Methodist Hospital, Radiation Oncology Department, 3535 Olentangy Road, Columbus, OH 43214*

*^7^ UCLA School of Medicine, Radiation Oncology Department, 200 UCLA Medical Plaza, Suite B265, Los Angeles, CA 90095*
